# Self-perceived recovery and quality of life in elderly patients surviving ICU-admission for abdominal sepsis

**DOI:** 10.1177/08850666211052460

**Published:** 2021-11-10

**Authors:** Anne C.M. Cuijpers, Marielle M.E. Coolsen, Ronny M. Schnabel, Tim Lubbers, Iwan C.C. van der Horst, Susanne van Santen, Steven W.M. Olde Damink, Marcel C.G. van de Poll

**Affiliations:** 1 82246 Department of surgery - Maastricht University Medical Centre, , Maastricht, the Netherlands; 2 82246 Department of Intensive Care Medicine - Maastricht University Medical Centre, Maastricht, the Netherlands; 3 199236 School for Oncology and Developmental Biology (GROW) – Maastricht University, Maastricht, The Netherlands; 4 199236 Cardiovascular Research Institute Maastricht (CARIM) – Maastricht University, Maastricht, The Netherlands; 5 385783 School for Nutrition and Translational Research in Metabolism (NUTRIM) – Maastricht University, Maastricht, The Netherlands; 6 Department of General, 39058 Visceral and Transplantation Surgery - RWTH University Hospital Aachen, Aachen, Germany

**Keywords:** Elderly, ICU-admission, abdominal sepsis, functional outcome, health-related quality of life, shared decision-making

## Abstract

**Introduction:**

Concern for loss of physical performance and Health-Related Quality of Life (HRQoL) may raise doubts regarding the meaningfulness of an Intensive Care (ICU) admission in elderly patients. We evaluated self-perceived long-term recovery and satisfaction in elderly surviving an abdominal sepsis related ICU-admission and related this to objective measures of HRQoL.

**Methods:**

A cross-sectional survey study was performed in all ICU-survivors with age ≥70 admitted with abdominal sepsis. HRQoL, frailty and self-perceived long-term recovery were measured using the EQ-5D-3L, Groningen Frailty Indicator, and a self-developed questionnaire, respectively.

**Results:**

Of 144 patients admitted, 48 were alive at follow up (2.42 [0.92; 3.83] years), and 29 (60%) returned the survey. Eleven patients out of 29 (38%) recovered to baseline functioning, and reported higher HRQoL compared to unrecovered patients (0.861 [0.807; 1.000] and 0.753 [0.499; 0.779] respectively, p=0.005). Of the unrecovered patients, 53% were satisfied with their functioning, and 94% were willing to return to ICU.

**Conclusions:**

Mortality in elderly patients with abdominal sepsis is high and ICU-admission should be weighed carefully. However, despite substantial functional decline in survivors, it does not necessarily cause self-perceived unsatisfactory functioning, poor HRQoL and unwillingness to receive life-sustaining therapy again. Caution is advised to use an anticipated loss of functioning as an argument to deny an ICU-admission.

## Introduction

A growing number of elderly patients is being admitted to the Intensive Care Unit (ICU) as a consequence of the increasing lifespan in the general population.^1^ The outcome of an unplanned ICU-admission in elderly is often poor, especially after sepsis caused by abdominal aetiologies.^2,3^ Abdominal sepsis mainly develops due to bowel perforation, anastomotic leakage, ischemic necrosis or other gastrointestinal tract injuries disrupting the integrity of the peritoneum and infecting the peritoneal cavity, but can also develop due to spontaneous bacterial peritonitis, mainly affecting cirrhotic or immunocompromised patients.^4^ Regardless of the cause of the abdominal sepsis, elderly requiring ICU-admission due to abdominal sepsis show a high ICU and in-hospital mortality rate, and elderly who survive up to hospital discharge often do so at the cost of functional impairment.^5,6^

The goal of admitting patients to the ICU is increasingly shifting from mere survival, to preserving an acceptable level of functioning and quality of life.^6^ Most current ICU research focuses on short-term outcome measures including ICU and in-hospital mortality or survival within the first year after hospital discharge. However, there is increasing recognition that the functional outcome and health-related quality of life (HRQoL) are perhaps equally important outcomes of an ICU treatment.

As recommended in ICU-admission guidelines, the decision to withhold ICU treatment in elderly patients should be based on physiological status, including the patient's life expectancy, patient's wishes, the likelihood of treatment success, and anticipated HRQoL after discharge, rather than on age per se.^7^

Sepsis is a condition associated with newly developed comorbidities and frailty.^8^ Therefore, knowledge on functional recovery and HRQoL in elderly ICU survivors is important to guide intensivists in shared decision-making, and counselling future patients to decide whether to be admitted to the ICU.^9^ Only little attention has been paid to the long-term functional recovery in elderly abdominal sepsis survivors, particularly their self-perceived recovery and HRQoL.

The aim of this study was to evaluate self-perceived long-term recovery and subsequent satisfaction in elderly surviving an abdominal sepsis-related ICU-admission, and to relate this to objective measures of HRQoL.

## Material and methods

### Study design

This study was a cross-sectional survey study conducted at the Maastricht University Medical Centre (MUMC+), a tertiary referral centre in the Netherlands, and reported using the Checklist for Reporting of Survey Studies (CROSS).^10^ Ethical approval was obtained by the local Medical Ethical Committee of the MUMC+ (METC 2017-0279).

### Data collection

All patients with age over 70 with an unplanned ICU-admission due to abdominal sepsis with either surgical and non-surgical aetiologies were retrospectively selected from a prospectively recorded database containing all patients admitted to the ICU with sepsis between 2012 and 2017. Any ICU-admission due to infection with at least one organ dysfunction was defined as admission with sepsis.^11^ Demographic, clinical and short-term outcome data (e.g. age, gender, pre-admission activities of daily living (ADL), comorbidities, reason for admission and length of ICU and hospital stay) were routinely obtained from all patients admitted to the hospital and retrieved from the electronic patient files. Information on pre-admission ADL was reported using the Modified Katz Index of Activities of Daily Living (Katz-ADL).^12^ Charlson Comorbidity Index (CCI) was calculated to quantify premorbid comorbidities.^13^ Revised cardiac risk index (RCRI) and modified frailty index (mFI) were available at baseline to quantify premorbid cardiovascular risk and frailty.^14,15^ Acute Physiology and Chronic Health Evaluation (APACHE) IV scores at ICU-admission were collected from the Dutch National Intensive Care Evaluation (NICE).^16^

For this study, all patients who were alive at time of the retrospective patient selection (August 2018) were cross-sectionally identified from the database and determined the sample size. Information about the study including the informed consent form and the questionnaires were mailed as a hardcopy to all elderly abdominal sepsis survivors in September 2018. A reminder was sent 6 weeks later to those who did not respond. Both the informed consent form and the questionnaires were returned in an envelope provided. Written informed consent was collected from all participants.

### Subjective perception of long-term recovery

Subjective perception of long-term recovery was measured using a questionnaire that was developed internally by two authors (AC and MvdP). Patients were asked questions on a two point scale regarding recovery and return to pre-admission functioning after hospital discharge (*In your opinion, did your recover completely to your pre-admission level of functioning after your ICU-admission and hospitalization?*), satisfaction with their recovery (*Are you satisfied with your level of functioning after ICU and hospital admission?*) and willingness to return to ICU (*Now you have experienced an ICU-admission, would you be willing to be re-admitted to the ICU department if this would be necessary?*). Patients were asked to answer with “*yes*” or “*no*”. To gain a deeper insight why elderly were willing to return to the ICU or not, patients were given the opportunity to write comments motivating their choice.

### Health Related Quality of Life (HRQoL) and post-ICU frailty

HRQoL was assessed using the Dutch version of the EuroQol Five-Dimensional descriptive system (EQ-5D-3L). The EQ-5D-3L is one of the most used instruments to quantify HRQoL and consists of two parts: the EQ-5D-3L descriptive system and the EQ-5D visual analogue scale (EQ-VAS).^17^ The EQ-5D-3L descriptive system uses five dimensions to describe different aspects of health: mobility, self-care, usual activities, pain/discomfort and anxiety/depression. Study participants are asked to score each domain using a three-level Likert scale: no problems, some problems or extreme problems, labelled 1 to 3.^17^ The EQ-5D-3L descriptive system can be converted into a summary index using a formula that attaches nation specific values to each of the levels in the five domains. The Dutch EQ-5D-3L tariff was used to calculate these summary indexes, ranging from 0.071 to 1.00 with 1.00 indicating optimal HRQoL.^18^ The EQ-VAS asks participants to self-rate their HRQoL using a vertical 100-point visual analogue scale labelled with the endpoints “*best health you can imagine*” and “*worst health you can imagine*”. A higher score indicates a better HRQoL.^17^

Additionally, the HRQoL of the study participants was compared to the Dutch age- and gender-matched population norms available for both the EQ-5D-3L descriptive system and the EQ-VAS.^19^ The current study population consists of patients aged 70 or over. EQ-5D-3L population norms are published for multiple age categories including age category 65-74 and 75+. Because the study population contains patients in both age categories, population norms for both categories are shown.

Post-ICU frailty was measured using the Groningen Frailty Indicator (GFI), a short 15-item questionnaire measuring loss of function in physical, cognitive, social and psychological domains. The GFI score ranges from 0 to 15 with a score of 4 or higher indicative of frailty.^20,21^

### Statistical analysis

Due to the small sample size, values were assessed as non-normally distributed and displayed as median with interquartile range [IQR] or as absolute number with percentages. Statistical analysis was performed using χ^2^ and Fisher exact test for categorical values and Mann–Whitney *U* tests for continuous variables. Two-tailed p<0.05 were considered statistically significant. Kaplan-Meier curves were used to visualise survival rates. Statistical analysis was performed using SPSS 26 (IBM Corp. Armonk, NY).

## Results

### Patients and baseline characteristics

Between 2012 and 2017, 144 patients had an unplanned ICU-admission due to abdominal sepsis. Seventy-two patients (50%) did not survive up to hospital discharge. An additional 17 (24% of the hospital survivors) died within the first year after ICU-admission. Seven more patients died later during follow up. Baseline characteristics of deceased patients are presented in Supplementary Table A, showing a higher APACHE IV score in patients not surviving up to hospital discharge.

**Figure 1.**
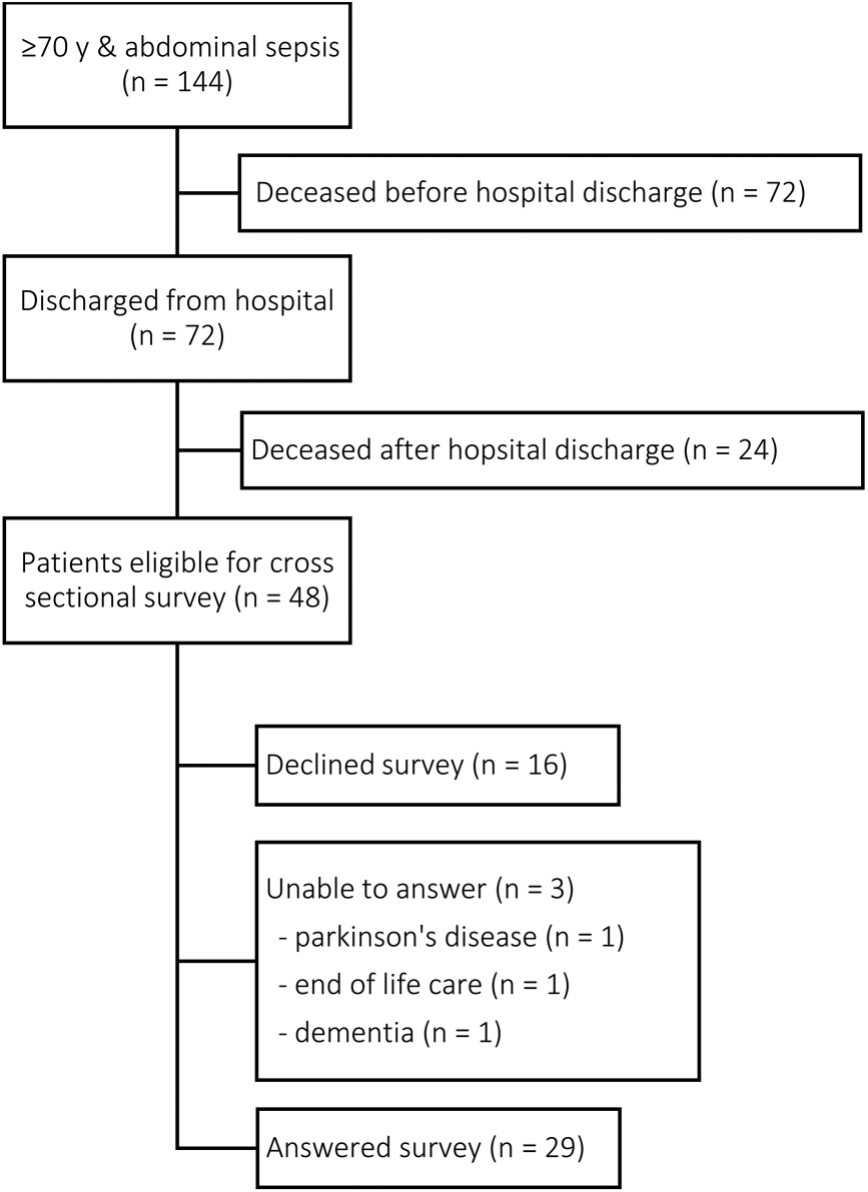
Patient inclusion flow chart

**Figure 2.**
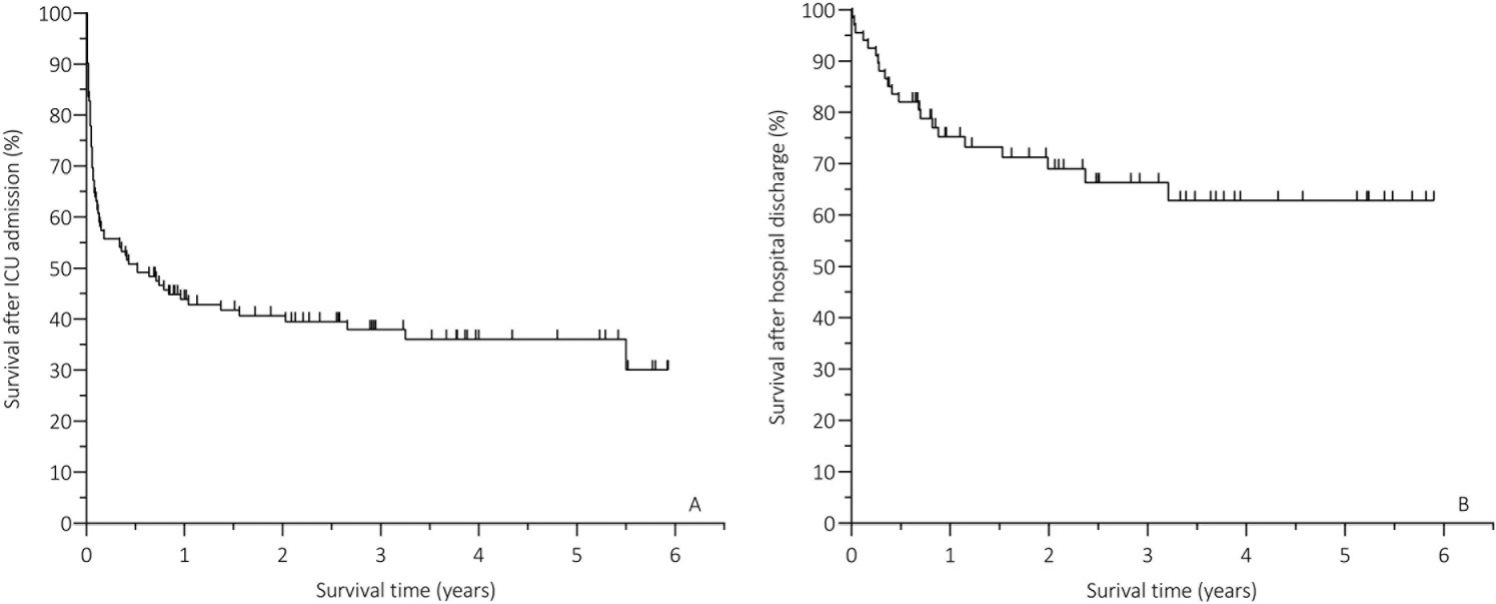
Survival rates: (A) overall survival from ICU-admission onwards; (B) survival after hospital discharge

The remaining 48 ICU survivors (median follow up time of 2.4 [0.9; 3.8] years) were asked to participate and were sent the survey. Sixteen did not return the survey for unknown reasons. Family members of three patients contacted the research team and reported that their relatives were unable to participate due to their current health state. Twenty-nine out of 48 patients (60%) returned the survey and were included in the study. The inclusion flowchart is shown in Figure 1. Overall survival rate from ICU-admission onwards, and survival rate of the patients who survived up to hospital discharge are visualised in Figure 2a and 2b, respectively. Median age was 75 [72; 81] and 73% of the participants were male. The majority of the patients were admitted postoperatively and suffered from bowel perforation, bowel obstruction, biliary tract infections or anastomotic leakages. Median length of ICU stay was 4 days with a broad interquartile range. Besides a shorter median follow up time in non-participants (1.8 [0.8; 3.3] years), baseline characteristics of participants did not differ from patients who did not participate in the survey (Table 1).

**Table 1.** Baseline characteristics.

	Total population N = 48	Responders N = 29	Non-responders N = 19	*p*-value
**Age (years)**	75 [72; 81]	75 [72; 79]	76 [74; 81]	0.153
**Gender**				
Male	35 (73%)	23 (79%)	12 (63%)	0.218
Female	13 (27%)	6 (21%)	7 (37%)	
**Admission type**				0.733
Elective surgery	13 (27%)	7 (24%)	6 (32%)	
Emergency surgery	25 (52%)	15 (52%)	10 (53%)	
Medical	10 (21%)	7 (24%)	3 (16%)	
**KATZ index**				0.396
0	36 (75%)	22 (76%)	14 (74%)	
1	4 (8%)	3 (10%)	1 (5%)	
3	1 (2%)	1 (3%)	0	
4	5 (10%)	3 (10%)	2 (11%)	
5	2 (4%)	0	2 (11%)	
**Charlson Comorbidity Index**	2.0 [1.0; 4.0]	3.0 [1.5; 5.0]	2.0 [0.75; 3.0]	0.137
APACHE IV*	78 [61; 91]	79 [61; 88]	74 [56; 97]	0.648
**Cause of sepsis**				0.654
Bowel perforation^1^	13 (27%)	6 (21%)	7 (36.8%)	
Bowel obstruction^2^	10 (21%)	6 (21%)	4 (21.1%)	
Biliary track infections	7 (15%)	5 (17%)	2 (10.5%)	
Anastomotic leakage	6 (13%)	4 (14%)	2 (10.5%)	
Fistula / intra-abdominal abscesses^3^	4 (8%)	3 (10%)	1 (5.3%)	
Biliary complications	3 (6%)	1 (3%)	2 (10.5%)	
Pancreatitis	2 (4%)	1 (3%)	1 (5.3%)	
Peritonitis of other cause^4^	3 (6%)	3 (10%)	0	
**Length of stay ICU (days)**	4 [2; 10]	4 [3; 13]	3 [2; 6]	0.305
Length of stay hospital (days)**	34 [22; 80]	31 [19; 69]	38 [22; 82]	0.569
Length of follow up (years)**	2.4 [0.9; 3.8]	2.6 [1.8; 4.1]	1.8 [0.8; 3.3]	***0.049***

Data displayed as absolute number (%) and median [IQR]. * N=39 (responders N=23, non-responders N=16); ** N=47 (non-responders N=18).

1: Both iatrogenic and spontaneous perforation based on infection or malignancy. 2: Obstruction based on adhesions, malignancy of volvulus. 3: Abscess and/or fistula postoperative or in combination with malignancy or chronic bowel disease. 4: Peritonitis in combination with chemotherapy or immune suppressant therapy.

Abbreviations: ADL: Activities of Daily Living, APACHE: Acute Physiology And Chronic Health Evaluation, ICU: Intensive Care Unit

### Subjective perception of long-term recovery

Eleven out of 29 survey responders (38%), who represented 8% of all patients with abdominal sepsis admitted to the ICU during the study period, indicated a recovery of self-perceived level of functioning comparable to before ICU-admission (baseline). There were no differences in baseline characteristics between patients who reached full recovery and patients who did not (see Table 2).

**Table 2.** Characteristics of subjectively recovered and not recovered elderly sepsis survivors.

	Recovered to baseline functioning N = 11	Not recovered to baseline functioning N = 18	*p*-value
Age (years)	75 [71; 80]	74 [72; 78]	0.928
Gender			0.326
Male	10 (91%)	13 (72%)	
Female	1 (9%)	5 (28%)	
KATZ index			0.120
0	10 (91%)	12 (67%)	
1	0	3 (17%)	
2	0	0	
3	1 (9%)	0	
4	0	3 (17%)	
ADL dependent	1 (9%)	6 (33%)	0.202
Modified frailty index	4.0 [3.0; 5.0]	4.0 [2.50; 5.0]	0.600
Charlson comorbidity score	3.0 [2.0; 4.0]	3.0 [1.0; 6.3]	0.616
Revised cardiac risk index	2.0 [1.74; 3.0]	2.0 [1.3; 3.0]	0.945
Apache IV*	73 [43; 88]	79 [62; 91]	0.301
Length of ICU stay (days)	4 [2; 14]	4 [2; 12]	0.538
Length of hospital stay (days)	45 [16; 80]	31 [20; 74]	0.875
Length of follow up (years)	3.7 [2.3; 5.4]	2.5 [1.5; 3.9]	0.234

Data displayed as absolute number (%) and median [IQR]. *N=23.

Abbreviations see legend Table 1.

As displayed in Table 3, 20 patients (71%) reported to be satisfied with their current level of functioning, including nine (53%) who reported no complete recovery. In addition, all patients who recovered to baseline functioning and 16 out of 17 patients (94%) who did not recover to baseline functioning expressed their willingness to undergo a new ICU-admission if needed. Table 4 presents written motivations of patients regarding a possible return to ICU.

**Table 3.** Subjective reported long-term recovery and health-related quality of life.

	Total population responders N = 29	Recovered to baseline functioning N = 11	Not recovered to baseline functioning N = 18	*p*-value
Satisfaction with recovery*				***0.010***
Yes	20 (71%)	11 (100%)	9 (53%)	
No	8 (27%)	0	8 (47%)	
Willingness to return to ICU**				0.458
Yes	25 (96%)	9 (100%)	16 (94%)	
No	1 (4%)	0	1 (6%)	
**Health related quality of life**				
EQ5D3L	0.775 [0.587;0.877]	0.861 [0.807; 1.000]	0.753 [0.499; 0.779]	***0.005***
EQ-VAS	60.0 [50.0;72.5]	75.0 [60.0; 80.0]	60.0 [50.0; 70.0]	0.071
**Difficulties in EQ-5D-3L domains**				
Mobility	23 (79%)	7 (64%)	16 (89%)	0.109
Self-care	10 (35%)	0 (0%)	10 (56%)	***0.003***
Usual activities	17 (59%)	3 (27%)	14 (78%)	***0.007***
Pain	16 (55%)	5 (46%)	11 (61%)	0.529
Anxiety	8 (28%)	1 (9%)	7 (39%)	0.087
**Post-ICU frailty**				** **
GFI*	5.0 [1.0;7.0]	1.0 [1.0; 5.0]	6.5 [3.8; 8.0]	***0.018***
Frail (GFI ≥4)*	18 (62%)	4 (36%)	14 (78%)	***0.048***

Data displayed as absolute number (%) and median [IQR]. Abbreviations: GFI: Groningen frailty indicator, other abbreviations see legend Table 1.

*N=28; **N=26; ***Determined as the percentage of participants who reported some or severe problems within the separate HRQoL domains as measured in the EQ-5D-3L.

**Table 4.** Motivation regarding willingness to return to the ICU, as reported by survivors*.

**Willing to return to ICU**
***Recovered to baseline functioning*** “In ICU, I regained substantial quality of life.” “You’re well taken care of.” “They are professionals and you receive the greatest attention.” “I received excellent care.”
***Not recovered to baseline functioning*** *“When it can save my life and give me a decent life afterwards”* *“In spite of everything, I want to continue to live in the best possible way.”* *“When I need critical care, I have no choice, it's the doctor's decision.”* *“They saved my life.”* *“I want to be helped.”* *“You’re in safe and capable hands.”* *“I want to get help when I’m seriously ill.”* *“I still want to live.”* *“When necessary, I still want to be treated to the utmost.”*
**Not willing to return to ICU**
***Not recovered to baseline functioning***
“I don't want to undergo surgery anymore.”

* N=26.

### Subjective recovery, Health Related Quality of Life and post-ICU frailty

**Figure 3.**
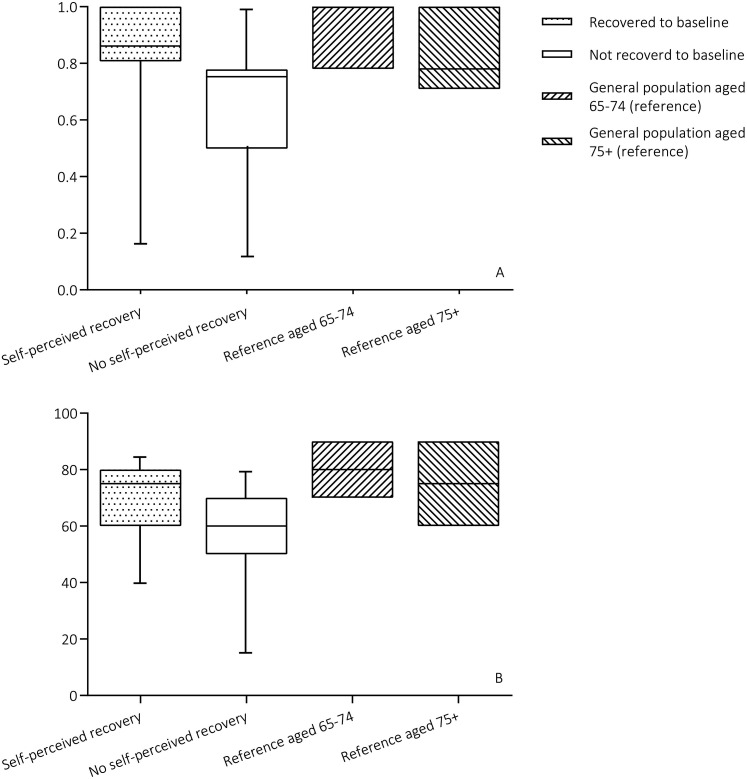
Health-related quality of life, compared to age- and gender matched reference population. A: EQ-5D-3L summary index scores. B: EQ-VAS scores.

The overall median score for HRQoL was 0.775 [0.587;0.877], which is lower compared to the matched reference population in the 65 to 74 age category (1.00 [0.87; 1.00]), but comparable to the reference population in the 75+ age category (0.78 [0.71; 1.00]) (Figure 3). Patients who recovered to baseline functioning had a significantly higher median HRQoL score compared to patients who did not recover to baseline functioning (0.861 [0.807; 1.000] and 0.753 [0.499; 0.779] respectively, p=0.005) (Table 3). The EQ-VAS showed an overall score of 60.0 [50.0; 72.5] with a trend toward higher score in patients who recovered to baseline functioning compared to those who did not (75.0 [60.0; 80.0] and 60.0 [50.0; 70.0] respectively, p=0.071). The EQ-VAS of recovered patients was comparable to the score of the 75+ aged reference population where overall EQ-VAS and the score of unrecovered patients were lower (EQ-VAS ref. aged 65-74: 80 [70; 90], ref. aged 75+: 75 [60; 90]) (Figure 3).

**Figure 4.**
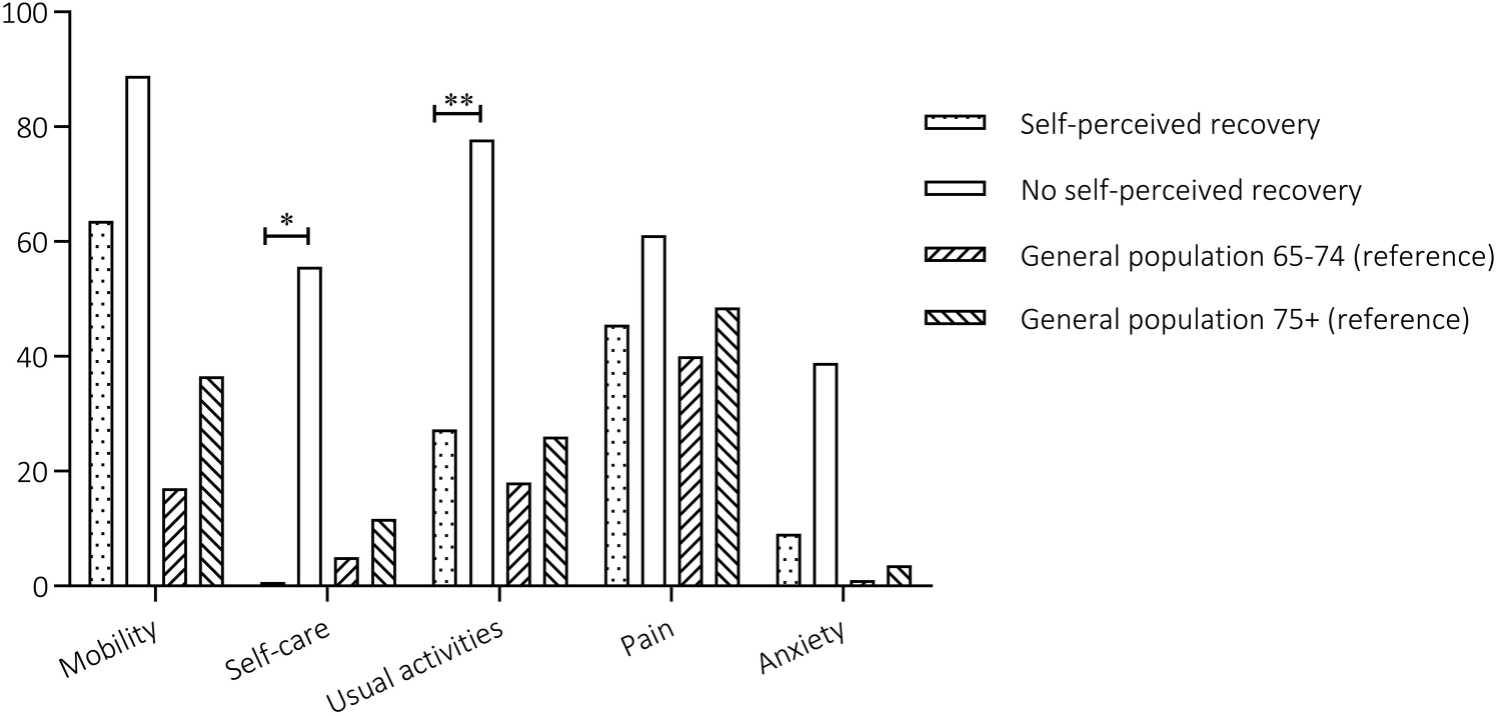
Percentage of patients reporting any problem within the separate EQ5D domains, * p=0.003, ** p=0.007.

Patients who did not return to their baseline functioning reported more difficulties in the assessed HRQoL domains (Table 3). The prevalence of problems with self-care was significantly higher compared to recovered patients (56% versus 0% respectively, p=0.003). Problems with usual activities (e.g. work, study, housework, and family of leisure activities) were also significantly more prevalent in patients who did not reach complete recovery compared to recovered participants (78% and 27%, respectively p=0.007). Difficulties with mobility where highly prevalent in both patient categories (64% in recovered and 89% in unrecovered patients). Unrecovered patients reported more problems in all HRQoL domains compared to the age and gender matched reference population. This patient category reported up to three times more problems with usual activities compared to the reference 75+ age category (78% and 26%, respectively). Problems with self-care were five times more prevalent (56% in unrecovered patients versus 11.7% in the 75+ age reference category), and problems with anxiety up to ten times more prevalent (39% in unrecovered patients versus and 4% in the 75+ age reference category). In the self-perceived recovered population, the HRQoL domains were comparable to the reference population except for the mobility domain (Figure 4).

Geriatric frailty was significantly more prevalent in the patients who not recovered to baseline functioning compared to recovered patients (78% and 36% respectively, p-value 0.048; Table 3).

## Discussion

This study showed that in-hospital and one-year mortality in elderly with a sepsis related ICU-admission is high. Elderly patients who survived an unplanned ICU-admission due to abdominal sepsis often did not recover to their pre-ICU level of functioning. Patients who did not recover to baseline functioning showed a lower satisfaction with their level of functioning and HRQoL compared to the recovered patients. Still, over half of the patients that reported incomplete self-perceived recovery were satisfied with their current level of functioning. Moreover, all but one survivor indicated their willingness to undergo another ICU-admission if needed. Mortality in elderly patients with abdominal sepsis is high and the decision to admit elderly to the ICU should be weighed carefully. However, although functional decline may be substantial among survivors, current results imply that this does not necessarily lead to a self-perceived unsatisfactory functioning, poor quality of life and unwillingness to receive life-sustaining therapy again.

These results add to the existing literature about long-term outcomes in elderly who survive a sepsis-related ICU-admission. Elderly surviving an unplanned ICU-admission often do not recover to baseline functioning and elderly who regain their self-perceived pre-ICU level of functioning often need over a year to do so.^22, 23^ However, previous literature showed that overall HRQoL in elderly surviving an ICU-admission remained satisfactory to good when compared to HRQoL prior to ICU-admission or to age matched general populations, regardless of the length of stay in the ICU.^23–28^ The current study differentiated between patients who experienced complete self-perceived recovery and those who did not, and showed difference in HRQoL in favour of the completely recovered patients. However, based on the documented satisfaction with recovery, and the motivations of the included participants showing their willingness to undergo life-sustaining treatment again, it is doubtful if the current HRQoL measurement provides a reliable reflection of all the items determining HRQoL in elderly.

Before ICU-admission, the willingness of elderly to receive life-sustaining treatment depends on the likelihood of beneficial outcomes, and a decline in functional status or loss of independency is often considered unacceptable.^6^ This makes physical functioning a leading factor in the decision-making towards an ICU-admission. Previous literature suggests that over 70% of the elderly would refrain from life-sustaining treatment when it would lead to decreased physical functioning afterwards.^29^ Based on current results, patients who actually experienced functional decline after an ICU-admission did not object future life-sustaining treatment. As shown, patients hold on to life, despite a decrease in functioning or objectively measured HRQoL. It seems that elderly patients modify the amount of functional decline that they find acceptable after having faced unexpected critical illness, and other values of HRQoL might become more important.^6^ Having experienced and survived an ICU-admission might even lead to a more positive view on a second ICU-admission compared to patients who did not experience an ICU-admission.^6^

To guide the shared-decision making whether or not to admit elderly to the ICU, and to counsel elderly and their relatives in the decision towards life-sustaining treatment, multiple factors are important to consider. Mortality after a sepsis-related ICU-admission remains high in elderly, both in-hospital and in the first year after discharge.^30, 31^ Next to the chances of survival and the benefit and harms of the intended treatment, personal thoughts of the patients regarding chances of survival, functional performance, acceptance of possible functional decline and loss of independency and quality of life should be discussed before admission.^9, 32^ However, reliable individual risk prediction of ICU outcome is lacking, especially regarding long-term recovery and functional outcome.^29^ Quality of life, frailty and functional status prior to life-sustaining treatment are not routinely measured in current practice. Despite no differences in frailty and premorbid functioning were observed in this study, previous literature has shown that these parameters might give a better indication of the expected short- and long-term outcome.^33–35^ Nevertheless, prior to an ICU-admission, it remains difficult to predict what a decline in physical functioning will mean to the patient, and how they cope with it. Using an anticipated loss of function and a possible decrease in objectively measured HRQoL in the decision to forego an ICU-admission in elderly patients should be handled with great caution, as a decrease in function or HRQoL does not necessarily lead to a self-perceived unsatisfactory life.

Current results are based on a highly selected population of elderly surviving an abdominal sepsis related ICU-admission and might be biased by selection. Elderly who are admitted to the ICU might already be selected due to triage before ICU-admission. Highly comorbid patients or patients with poor functional or mental status might refuse ICU-admission or might be refused on medical grounds leading to possible selection bias in the current study. Furthermore, it was unknown why 33% of the potential participants did not return the survey. Despite no significant differences in baseline characteristics between responders and non-responders, long-term outcome of these patients including potential physical or cognitive decline impeding participation remained unknown introducing another possible source of selection bias. Further limitations affecting generalizability of the results were the small sample size and single centre study design. In addition, the study population was predominantly male and the majority of the participants suffered from surgery related abdominal sepsis aetiologies. It is unknown if sex or the cause of abdominal sepsis affect a patient's long-term self-perceived recovery and HRQoL. Due to the retrospective nature of this study, no baseline HRQoL and GFI measurements were available. Baseline mFI was collected retrospectively, but this instrument mainly focusses on comorbidities and is therefore a less reliable frailty measurement. Future studies should focus on how to obtain a reliable multidimensional patient profile before ICU-admission which can be used for reliable individual risk prediction of both short and long-term ICU outcome.

## Conclusions

Mortality in elderly patients with abdominal sepsis requiring ICU-admission is high and most surviving patients will experience long lasting disabilities. However, functional decline does not necessarily lead to an unsatisfactory functioning, poor quality of life and unwillingness to receive life-sustaining therapy again in patients surviving an abdominal sepsis related ICU-admission. These results suggest that elderly might adjust what level of functioning they find satisfactory when faced with the sequelae of critical illness. Anticipated loss of functional outcome, and lost quality of life should be used with caution as an argument in shared decision-making regarding ICU-admission in elderly sepsis patients.
